# Wnt3 and Wnt3a are required for induction of *the mid*-*diencephalic organizer *in the caudal forebrain

**DOI:** 10.1186/1749-8104-7-12

**Published:** 2012-04-04

**Authors:** Benjamin Mattes, Sabrina Weber, João Peres, Qing Chen, Gary Davidson, Corinne Houart, Steffen Scholpp

**Affiliations:** 1 Karlsruhe Institute of Technology (KIT), Institute of Toxicology and Genetics (ITG), Karlsruhe, Germany; 2 MRC Centre for Developmental Neurobiology, King's College London, London, UK

**Keywords:** Forebrain patterning, Thalamus development, Zona limitans intrathalamica, ZLI

## Abstract

**Background:**

A fundamental requirement for development of diverse brain regions is the function of local organizers at morphological boundaries. These organizers are restricted groups of cells that secrete signaling molecules, which in turn regulate the fate of the adjacent neural tissue. The thalamus is located in the caudal diencephalon and is the central relay station between the sense organs and higher brain areas. The mid-diencephalic organizer (*MDO*) orchestrates the development of the thalamus by releasing secreted signaling molecules such as Shh.

**Results:**

Here we show that canonical Wnt signaling in the caudal forebrain is required for the formation of the Shh-secreting *MD *organizer in zebrafish. Wnt signaling induces the *MDO *in a narrow time window of 4 hours - between 10 and 14 hours post fertilization. Loss of Wnt3 and Wnt3a prevents induction of the *MDO*, a phenotype also observed upon blockage of canonical Wnt signaling *per se*. Pharmaceutical activation of the canonical Wnt pathways in Wnt3/Wnt3a compound morphant embryos is able to restore the lack of the *MDO*. After blockage of Wnt signaling or knock-down of Wnt3/Wnt3a we find an increase of apoptotic cells specifically within the organizer primordium. Consistently, blockage of apoptosis restores the thalamus organizer *MDO *in Wnt deficient embryos.

**Conclusion:**

We have identified canonical Wnt signaling as a novel pathway, that is required for proper formation of the *MDO *and consequently for the development of the major relay station of the brain - the thalamus. We propose that Wnt ligands are necessary to maintain the primordial tissue of the organizer during somitogenesis by suppressing Tp53-mediated apoptosis.

## Background

The thalamic complex consists of the anteriorly located pre-thalamus and the posterior located thalamus [1]. The prosomeric model would describe these two areas as main dorsal components of the prosomere 3 (P3) and prosomere 2 (P2) respectively [2]. Between these two neural segments there is an intervening ventricular ridge - the *zona limitans intrathalamica *(*ZLI*). The anatomical *ZLI *border zone contains a small cell population, which releases signaling molecules. This signaling center orchestrates thalamus development by controlled release of the morphogen Sonic hedgehog and thus, we termed it the *middiencephalic organizer *(*MDO*, formerly known as the *ZLI organizer*; [3]). Lack of the Shh-positive *MDO *leads to gross malformation of the caudal forebrain and loss of the entire thalamus. Local abrogation of Shh signaling in small cell clones blocks acquisition of thalamic neuronal cell fate in vertebrates [4-6]. Thus the *MDO *determines the size of the thalamic complex and orchestrates the neuronal development of the central relay station of the brain.

A further important diffusible, external cue during neural development is Wnt signaling. Patterning of the vertebrate anterior neural tube requires the function of this pathway at multiple stages [7]. Canonical Wnt signaling regulates anteroposterior patterning in the forebrain and midbrain, is required for development of the dorsal telencephalon [8] and the eyes [9], and allows the establishment of the midbrain-hindbrain boundary *(MHB) *organizer [10,11]. Several lines of experimental evidence have demonstrated that other signaling pathways counteract Wnt signaling during neural development, independent from direct antagonists, such as sFRPs or Dkk1. Indeed, Shh and Wnt signaling are mutually antagonistic during some events in embryonic development, such as spinal cord patterning [12]. Despite the recognized importance of Wnt signaling for central nervous system (CNS) development, its functional relevance during diencephalon formation and how Wnt signaling and Shh signaling interact there remains unknown. Receptors, ligands and modifiers of the Wnt signaling pathway are expressed during early stages of caudal forebrain regionalization [13,14]. Recently, we showed that Wnt signaling is required for cell adhesion in the thalamus and thalamic neurogenesis [15]. However, the early function of Wnts during *MDO *establishment has to be determined.

Here we show that blockage of the canonical Wnt signaling pathway leads to malformation of the *MDO*. By a Morpholino-based knock-down approach we identified Wnt3 and Wnt3a as the responsible ligands and hence are required to maintain the primordium of the *MDO*. Lack of canonical Wnt signaling *per se *or knock-down of Wnt3/Wnt3a leads similarly to an increase of apoptosis specifically within the organizer primordium. Consistently, blockage of Tp53-mediated apoptosis is able to rescue the *MDO*. Furthermore, abrogation of the repressive factors Fezf2 and Irx1b leads to restoration of the organizer. In summary, we propose that canonical Wnt signaling triggered by Wnt3/Wnt3a is necessary to suppress Tp53-mediated apoptosis and thus maintain the organizer tissue during development.

## Results

**Figure 1:**
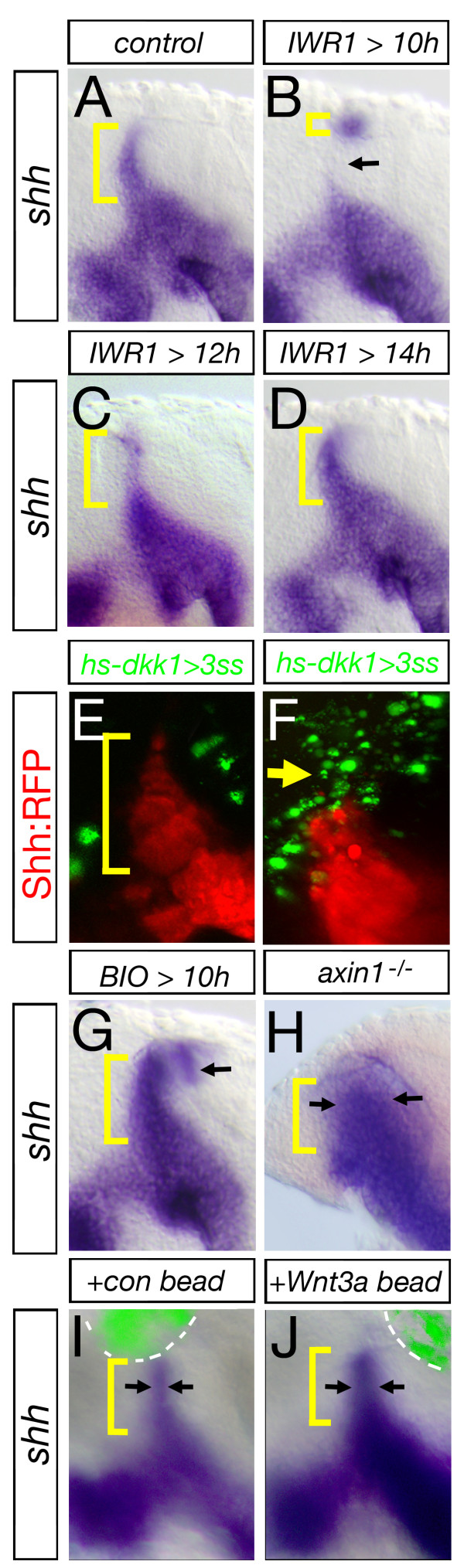
**Canonical Wnt signaling is required for induction of Shh expression in the *MDO***. *MD organizer *in lateral views of embryos with anterior to the left at 28 hpf. Blockage of canonical Wnt signaling by treatment of embryos with 40 μM IWR1 at different time points leads to the following alterations in the formation of the *shh *positive *MDO*: 10 to 28 hpf - strong decrease (B, arrow), 12 to 28 hpf - mild decrease (C) 14 to 28 hpf - no alteration (D). *Shh *expression at the MDO is unaltered if Dkk1-GFP positive cells are located in the telencephalon and the pretectum (E), however, the *shh *positive *MDO *is absent if Dkk1-GFP cells are located in the thalamic complex (F, n = 6/10, arrow). Ectopic activation of the Wnt signaling pathway by BIO treatment (G, n = 12/18), in the *axin1-/- *mutant embryos (H) as well as embryos with an implanted Wnt3a loaded bead (I, J, n = 3/5) leads to a increase of *MD organizer *shown by expanded s*hh *expression (black arrows).

Several members of the Wnt family are localized at or near the roof plate - the dorsal pole of the developing nervous system where they dorsalize the embryonic brain and spinal cord [16]. In the latter, canonical Wnt signaling is opposed by the ventralizing Shh signal [17]. A similar scenario has been described for the telencephalon in which *wnt8b *expression is restricted by the Shh-dependent transcription factor FoxG1 [8]. To test whether canonical Wnt signaling is required for the formation of the Shh-positive *MDO*, we treated embryos with a specific Wnt signaling antagonist, the tankyrase inhibitor IWR1 [18]. We used IWR1 to block Wnt signaling from 10 to 28 hpf, after initial neural plate patterning has taken place. Under these conditions, we observed a strong reduction of *Shh *in the central part of the *MDO*, whereas expression at the dorsal tip remained (Figure 1A, B). Initiating treatment at 12 hpf led to a mild decrease whereas treatment after 14 hpf did not affect *shh *expression in the *MDO *(Figure 1C, D), suggesting that canonical Wnt signaling is required for *MDO *induction in a narrow time window of four hours. We used a heat-shock inducible Dkk1-GFP construct as an additional tool to inhibit Wnt signaling [19]. After activation of Dkk1-GFP at 10 hpf, we found a lack of Shh::RFP expression within the *MDO *at 28 hpf (Figure 1E, F). Consistently, enhancement of Wnt signaling between 10 and 28 hpf using the GSK3ß inhibitor BIO [20] led to a broader expression domain of *shh *at the *MDO *(Figure 1G). In agreement, *shh *expression is expanded in the diencephalon of *axin1^-/- ^*mutant embryos [21], independent of morphological alteration of the telencephalon (Figure 1H). Similarly, grafting Wnt3a loaded beads in the diencephalon at 10 hpf (Figure 1I) resulted in expansion of *shh *expression at the *MDO*, compared to embryos implanted with PBS-loaded control beads. Therefore, we hypothesize that canonical Wnt signaling is necessary for proper induction of the *MDO *between 10 h and 14 hpf.

**Figure 2:**
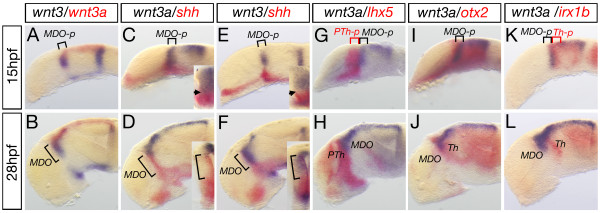
**Wnt3 and Wnt3a mark the *MDO* and the dorsal thalamus**. Analysis of expression dynamics of indicated marker genes at 15 and 28 hpf. At 15 hpf, *wnt3 *and *wnt3a *are expressed in the *MDO primordium (MDO-p) *and maintained in the organizer at 28 hpf. Cross section analysis of the left hemisphere reveals that *wnt3a *expression is adjacent to *shh *expression at the ventricular zone of the organizer (D), whereas *wnt3 *expression co-localizes with *shh *expression at 28 hpf (F). In the diencephalon, expression of *wnt3a *is located between the *lhx5 *expression domain (G, H) and the *irx1b *expression domain (K, L). Consistently, *wnt3a *is co-expressed with *otx2 *(I, J) suggesting that *wnt3a *marks the *MD organizer *territory at 15 hpf and 28 hpf. *MDO*, PTh, prethalamus, Th, thalamus, *-p, *-primordium.

Prime candidates to mediate local canonical Wnt signaling in the developing thalamus are Wnt3 and Wnt3a. At 15 hpf and 28 hpf, both of these Wnt ligands are expressed in the *MDO*, whereas *wnt3a *also marks the roof plate (Figure 2A, B). Wnt3 and Wnt3a are expressed in the mid-diencephalon prior induction of *shh *in the *MDO *at 15 hpf (Figure 2C, E). At 28 hpf, *shh *overlaps with *wnt3 *expression domain in the ventricular zone of the *organizer *(Figure 2F). However, expression of *wnt3a *becomes restricted to the dorsal *MDO *at 28 hpf and is subsequently localized subjacent to *shh *(Figure 2D). To address whether these two Wnt ligands mark the primordium of the *MDO*, we analyzed the expression pattern relative to markers in the thalamic complex. The pre-thalamic marker *lhx5 *[22] abuts the *wnt3a *expression domain anteriorly (Figure 2G, H). Wnt3a overlaps with the *MDO*/thalamus marker *otx2 *(I, J; [23]). The thalamic marker *irx1b *is expressed mainly posterior to the expression domain of *wnt3a *except of the most dorsal area (Figure 2K, L). Thus, *wnt3 *and *wnt3a *mark the primordium of the *MDO *prior to the induction of *shh *expression.

**Figure 3:**
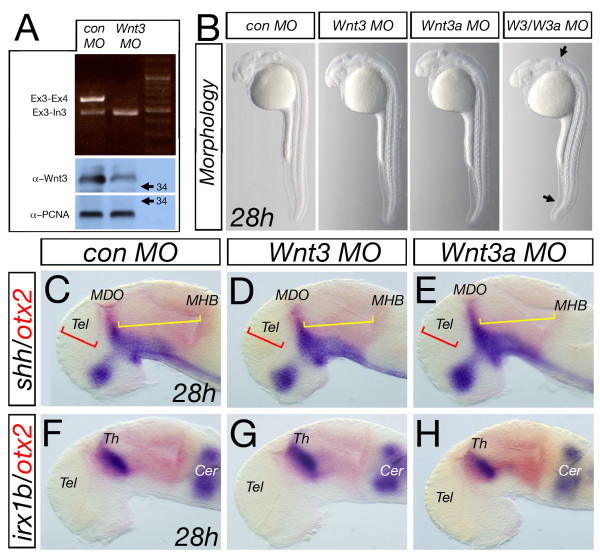
**Analysis of knock-down of Wnt3 and Wnt3a**. Injection of *wnt3 *MO leads to a decrease in splicing of mRNA at the exon3-exon4 boundary and an increase in non-spliced RNA product visualized by amplification at the exon3-intron3 boundary and subsequently to a reduction of Wnt3 protein in morphant embryos at 28 hpf (A). Knock-down of either Wnt3 or Wnt3a leads to an indistinguishable morphology to the control Morpholino-injected embryos whereas the double morphant embryos show a significant alteration in brain morphology, a kinked tail and a smaller otic vesicle (B, arrows). Similarly, analysis of marker genes in the forebrain midbrain area shows no alteration in control, *wnt3 *or *wnt3a *morphant embryos (C-H). Cer, cerebellum.

To elucidate the early function of Wnt3/Wnt3a during development of the thalamic complex, we blocked their function by performing a Morpholino antisense oligomere (MO)-based knock-down approach [24]. Injection of a splice-blocking *wnt3 *MO leads to a severe down-regulation of Wnt3 protein by Western blot analysis (Figure 3A). Brain morphology in embryos injected with either *wnt3 *MO or *wnt3a *MO is not visibly distinguishable from *control *MO injected embryos (Figure 3B-H). This suggests that Wnt3 and Wnt3a have redundant functions during brain development.

**Figure 4:**
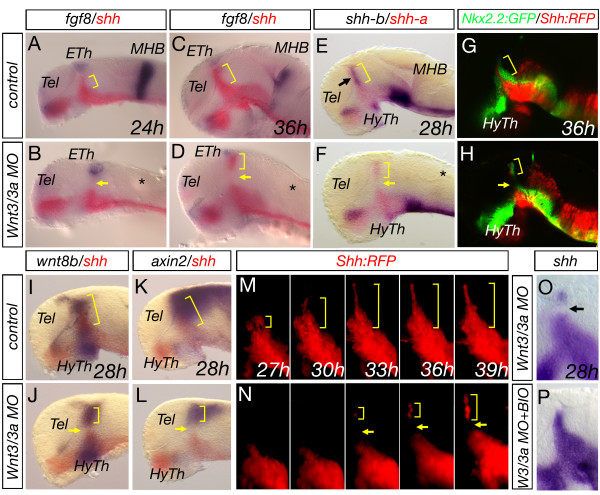
**Wnt3 and Wnt3a are required for *MDO* formation**. Knock-down of Wnt3 and Wnt3a leads to a down-regulation of the expression of indicated markers in the central part of the *MDO *(A-L). Time-lapse analysis of Shh::RFP shows an independent induction of the dorsal remaining organizer spot (M, N). Treatment of *wnt3/wnt3a *morphant embryos with the Wnt signaling agonist BIO from 10 to 28 h leads to a rescue of *shh *expression at the *MDO *(O, P). Brackets mark the extent of the *MDO*, whereas the arrows indicate the gap between the basal plate and the remaining spot of dorsal MDO identity. Eth, epithlamus, HyTh, hypothalamus, MHB, midbrain-hindbrain boundary, Tel, telencephalon.

However, combining MOs targeting Wnt3 and Wnt3a (*wnt3/wnt3a *MO) led to a strong decrease of the expression of the pan-*MDO *marker *shh *in the central part of the *MDO *at 24 and 36 hpf (Figure 4A-D) These results are comparable to global Wnt signaling inhibition between 10 and 28 hpf. (Figure 1A, B). A combinatorial requirement of canonical Wnt signaling during *MHB *organizer formation has been previously reported [11]. Indeed, the *MHB *organizer marked by *fgf8 *expression is lacking in double morphant embryos (Figure 4A-D, asterisks) thus validating the specificity of the *wnt3/wnt3a *MO approach. In addition to the altered *shh *expression (also known as *shh-a*), we find that the expression of the paralogue *shh-b *is absent in the organizer in compound morphant embryos (Figure 3E, F; black arrow). Down-regulation of *shh *and *shh-b *is accompanied with a down-regulation of the Shh-dependent target gene *nkx2.2*, (here *Nkx2.2::GFP*) adjacent to the *MDO *in *wnt3/wnt3a *morphant embryos (Figure 4G, H).

Next, we analyzed the expression of components of the Wnt signaling pathway, *wnt8b *and *axin2 *in *wnt3/wnt3a *morphant embryos. We observed a reduction of *wnt8b *expression in the central area of the *MDO *(Figure 4I, J). Consequently, the Wnt signaling target gene *axin2 *shows a reduced expression in the caudal forebrain (Figure 4K, L). These results suggest that Wnt3 and Wnt3a are required for proper *MDO *formation. All analyzed marker genes show a consistent alteration - the central area of the *MDO *shows a down-regulation, whereas the dorsal tip seems to be less affected by the knock-down and displays a residual robust expression of *MDO *markers such as *shh *and *wnt8b *(Figure 4D, H, J, L). We wondered whether *MDO *fate is induced within tip cells of compound morphant embryos or if cells from the basal plate migrate dorsally to form the *MDO *spot. Therefore, we performed a time-lapse analysis using the *Shh::RFP *transgenic zebrafish line with strong expression of the transgene in the basal plate prior *MDO *formation [25]. At 27 hpf, we detected *Shh::RFP *expression in the ventral *MDO *with a progressively dorsal expansion over the next 12 h (Figure 4M). In Wnt3/Wnt3a-deficient embryos we observed induction of dorsal *MDO shh *expression independently of basal plate contact (Figure 4N). This is in agreement with the so-called bucket-brigade induction model of the *MDO *[6]. In this model cells progressively adopt *MDO *fate from ventral to dorsal without changing their dorso-ventral position. Importantly, activation of the canonical Wnt pathway using the GSK3ß inhibitor BIO between 10 and 28 hpf is sufficient to restore *MDO *formation in Wnt3/Wnt3a deficient embryos (Figure 4O, P).

From the performed knock-down experiments, we conclude that canonical Wnt signaling between 10 and 14 hpf, by Wnt3/Wnt3a, is required for *shh *induction at the *MDO*.

**Figure 5:**
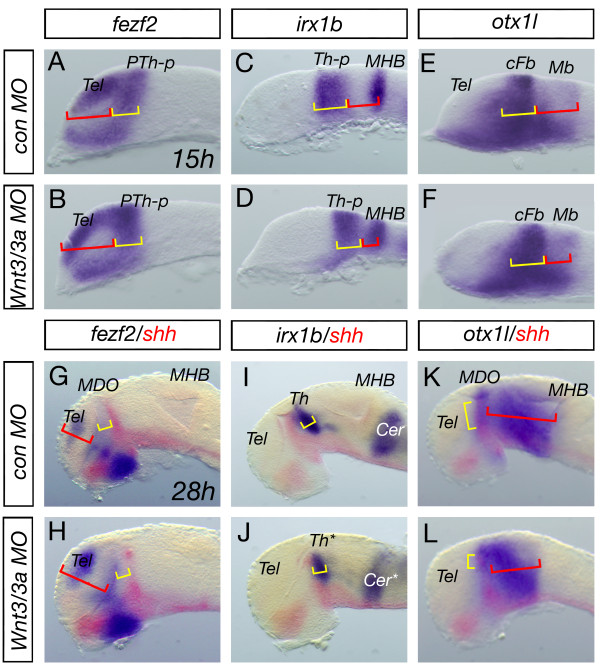
**Primordia of the prethalamus and the thalamus are not affected in Wnt3/Wnt3a morphant embryos**. Analysis of the *fezf2 *positive anterior forebrain area at 15 and 28 hpf reveals an expansion of the telencephalic domain (red brackets), however, the prethalamus (yellow brackets) is unaltered *wnt3/wnt3a *morphant embryos (A, B, G, H). Posteriorly analysis of the *irx1b *as well as the *otx1l *expression shows that the thalamic domain does not change significantly (yellow brackets), whereas, the midbrain territory shrinks in compound morphant embryos (red brackets, C-F; I-L). Notably, although both organizers - the *MDO *and *MHB *- disappeared in *wnt3/wnt3a *morphant embryos, expression of competence factors like *fezf2 *and *irx1b *are unaltered at 28 hpf, suggesting a specific function of Wnt3/Wnt3a in organizer formation. cFb, caudal forebrain, Mb, midbrain.

**Figure 6:**
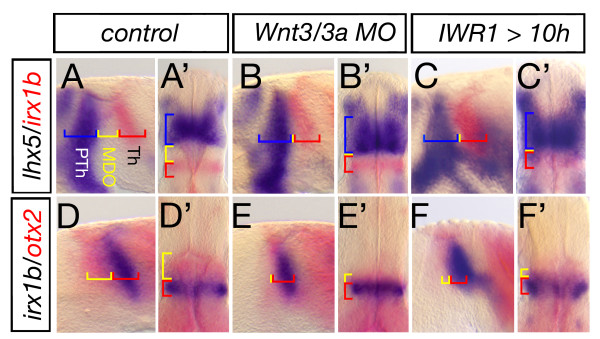
**Blockage of Wnt signaling lead to lack of *MDO* tissue**. Lateral views and dorsal views (marked by ') of embryonic heads at 28 hpf. *Wnt3/wnt3a *morphant embryos lack the organizer tissue and the *lhx5 *positive prethalamus (PTh, blue) abuts the *irx1b *thalamus (Th, red) (A-B', n = 54/78). A similar phenotype is observed in embryos treated with 30 μM IWR1 from 10 to 28 h to inhibit canonical Wnt signaling (n = 15/17). Hence, the *otx2 *positive *MDO *(yellow) is lacking if Wnt3/Wnt3a function is knocked-down (n = 44/80, D-E'), likewise after inhibition of canonical Wnt signaling (F, F', n = 14/24).

Next we studied the consequences of the knock-down of Wnt3/Wnt3a on thalamic regionalization. Therefore, we analyzed the expression pattern of the prethalamic marker *fezf2 *and the thalamic marker *irx1b *at 15 hpf and at 28 hpf (Figure 5A-D, 5G-J). We find that the size of expression domains of *fezf2 *and *irx1b *are unaltered in Wnt3/Wnt3a compound morphant embryos compared to the control embryos. In addition, in mouse and fish embryos with reduced Otx function, a similar lack of the *MDO *and of the thalamus has been noted [23,26]. We find that the expression of the forebrain/midbrain marker *otx1l *(Figure 5E, F, K, L) and of *otx2 *(Figure 6D, E') is reduced in the morphant forebrain and midbrain suggesting a similar functional connection between Otx and Shh in the compound morphant embryos.

To elucidate this aspect further, we mapped the expression of markers of the prethalamus relative to markers of the thalamus. Interestingly, we found the *lhx5 *positive prethalamus abuts the *irx1b *positive thalamus and the *MDO *anlage is lacking in compound morphant embryos at 28 hpf (Figure 6A-B'). Likewise, analysis of the *MDO *and thalamus primordia in these embryos showed that the *otx2*-positive *MDO *is severely decreased, whereas the size of the *irx1b *and *otx2*-positive thalamus is unaltered (Figure 6D-E'). This suggests that Wnt3/Wnt3a are required for establishment of the *MDO *primordium. Consistently, blockage of Wnt signaling with IWR1 between 10 and 28 hpf leads to a similar phenotype (Figure 6C, C', F, F'). This suggests that Wnt3/Wnt3a function is required to maintain the anlage of the *MDO*, but not for maintenance of the primordia of the prethalamus and thalamus.

**Figure 7:**
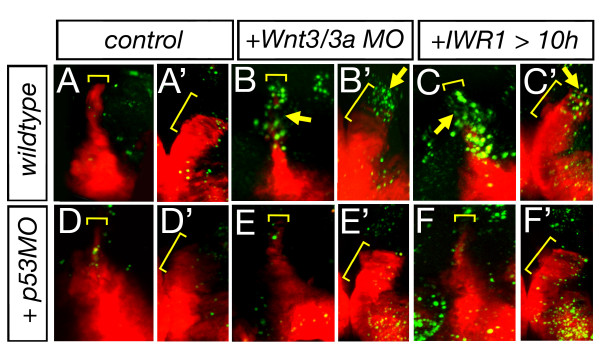
**Lack of Wnt signaling leads to apoptosis in the *MDO***. Lateral views and dorsal views of the left hemisphere (marked by ') of Shh::RFP transgenic embryos at 36 hpf. *Wnt3/wnt3a *morphant embryos as well as embryos treated with IWR1 display an increase of apoptotic cells within the *MDO *at 36 hpf (n = 4/6, A-C'). Upon blockage of Tp53 function apoptosis is down-regulated within the organizer tissue of *wnt3/wnt3a *morphant embryos as well as embryos treated with IWR1 and consistently, *shh:RFP *expression is restored (D-F').

We next considered the Wnt-mediated process that maintains the *MDO *anlage. Wnt signaling plays a pivotal role in cell survival [27] providing a possible explanation for the lack of the *MDO *primordium in Wnt3/Wnt3a deficient embryos. Therefore, we analyzed apoptosis by usage of the fluorescent cationic dye, acridine orange, which permeates dying cells to bind chromatin [28]. Indeed, compound morphant embryos display an accumulation of dying cells within the organizer primordium (arrow, Figure 7A-B'). Consistently, blockage of canonical Wnt signaling by treatment with IWR1 leads to a similar increase of apoptotic cells specifically within the organizer anlage (Figure 7C, C'). Consistently, blockage of Tp53 function is able to rescue *shh *expression in the organizer in Wnt3/Wnt3a morphant embryos as well as in IWR1 treated embryos (Figure 7D-F). This suggests that a main function of Wnt3/Wnt3a in the caudal forebrain is to ensure the survival of *MD *organizer cells.

**Figure 8:**
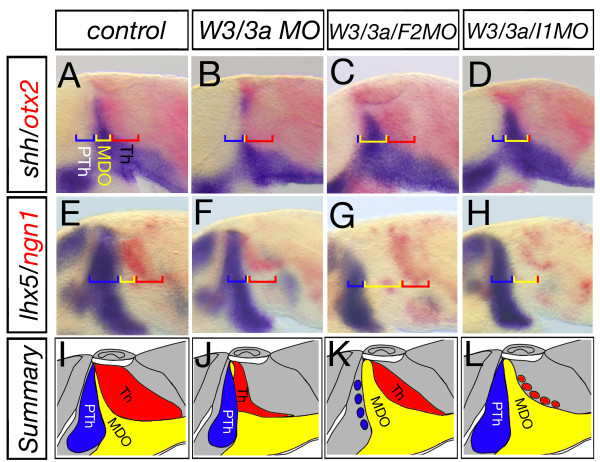
**FezF2 and Irx1b limit the *MDO* at the AP axis**. *Shh *expression in the *MDO *is decreased in Wnt3/Wnt3a deficient embryos (A, B, n = 45/55), whereas, prethalamic identity as well as thalamic identity is maintained (E, F, n = 18/23). In triple morphant embryos lacking Wnt3, Wnt3a and Fezf2 function, a restoration of *shh *expression is observed in the *MDO *(C, n = 28/31) accompanied by a reduction of *lhx5 *expression in the pre-thalamus (G, n = 10/34). In *wnt3/wnt3a/irx1b *triple morphant embryos, we observe a similar restore of the *shh *positive *MDO *(D, n = 28/31); however, here the *neurog1 *positive thalamus shrinks (H, n = 25/32, N, n = 20/25). For explanation of the summary (I-L) see the text.

Previous evidence has suggested that members of two transcription factor families, Fez [29] and Irx [30] are involved in induction of the Shh-positive organizer. However, recent observations show that prethalamic Fez limits the anterior border of the *MDO *[31], whereas the thalamic Irx genes are able to repress Shh expression at the posterior limit of the *MDO *[1,23]. Therefore, we asked if the main function of Wnt3/Wnt3a in the developing thalamus is the establishment of a Fez/Irx-free zone in the caudal forebrain to allow formation of the *MDO*. We first tested whether in this situation a similar reduction of *shh *is seen in embryos deficient for Wnt3/Wnt3a function (Figure 8A, B). As a broadening of the *MDO *in embryos deficient for FezF2 [31] and Irx1b [23] has been reported previously, we wondered if we would be able to restore the *MDO *in the individual triple morphant embryos. Indeed, upon simultaneous reduction of Fezf2 or Irx1b function, we observe a rescue of *shh *expression at the *MDO *of *wnt3/wnt3a *morphant embryos (Figure 8C, D). To elucidate the impact of the knock-down on the formation of the thalamic complex, we analyzed the expression of the pre-thalamic marker *lhx5 *and the thalamic proneural factor *neurog1*. In *wnt3/wnt3a/fezf2 *triple morphant embryos, the broadening of the *shh*-positive *MDO *is accompanied with down-regulation of the dorsal pre-thalamic *lhx5+ *domain, suggesting that the *MDO *expands at the expense of the pre-thalamus (Figure 8G). On the other hand, the *shh *expression domain at the *MDO *that is restored in the *wnt3/wnt3a/irx1b *morphant embryos is accompanied by a reduction of the *neurog1 *positive thalamus, presumably by expansion of the *MDO *into the thalamic territory (Figure 8H). We, therefore, conclude that both adjacent areas - prethalamus and thalamus - would be competent to act as *MDO*. However, Fez and Irx are required to repress organizer formation, to shape the overall domain of the *MDO *and to maintain the identities of adjacent brain regions (Figure 8I-L).

## Discussion

**Figure 9:**
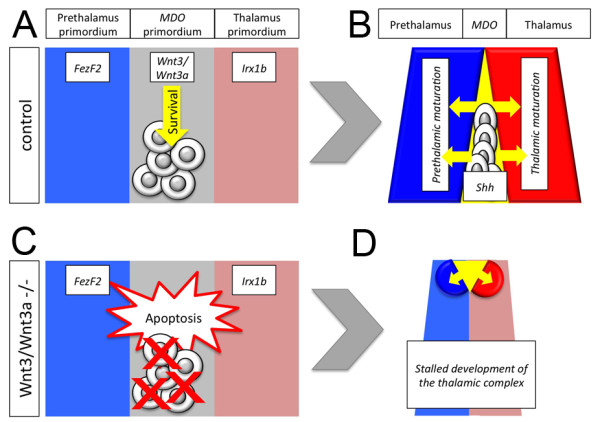
**Summary**. Wnt3 and Wnt3a signaling maintains the *MDO *primordium by protecting the cells from apoptosis (A). The mature *MDO *secrets organizer signals, such as Shh, which drives thalamic development (B). In Wnt3/Wn3a deficient embryos, *MDO *cells go into apoptosis (D). Loss of the organizer leads to stalled development of the thalamic complex (D).

Here we have described a new aspect of Wnt signaling during caudal forebrain development. Wnt3 and Wnt3a mark the *MDO *prior to the expression of the principal thalamus organizing signal Shh. Blockade of Wnt signaling leads to the lack of the *MDO *tissue (Figure 9). We show that cells of the organizer primordium go into Tp53-mediated apoptosis upon loss of the Wnt stimulus. We hypothesize that the main function of Wnt3/Wnt3a-mediated signaling in the organizer is to protect cells from cell death as blockage of Tp53-mediated apoptosis is able to restore the organizer function. Thus, Wnt3/Wnt3a-mediated signaling is the survival factor for organizer cells only, but not for the surrounding thalamic complex.

### Wnt3 and Wnt3a are the principal, but not sole Wnt ligands during *MDO* formation

In Wnt signaling-deficient embryos, we find a persistent spot of Shh-positive cells in the dorsal most tip of the organizer. There are two possibilities to explain this phenotype. First, the dorsal diencephalic roof plate is a rich source of several Wnt ligands: in addition to Wnt3a, we find expression of Wnt8b, Wnt1 and others suggesting that there is a compensation mechanism operating at the dorsal *MDO*. Indeed, in a few embryos treated with IWR1 or overexpressing the Wnt antagonist Dkk1 (Figure 1), we observed a total block of organizer formation. However, these treatments also led to gross malformation of the embryo making it difficult to identify a specific Wnt-related function. A further explanation could be the third signaling pathway important for thalamus formation, the Fgf pathway. Fgf ligands, such as Fgf8, are strongly expressed at the dorsal area of thalamic anlage - in the epithalamus. Here, Fgf signaling is required for the formation of the rostral thalamus and influences expression of thalamic transcription factors such as Gbx2 [32,33]. This could suggest that Fgf signaling is required independently to maintain *MDO *fate, a possibility that requires future analysis.

### Wnt signaling during thalamus development

Wnt signaling is important to set up the initial anteroposterior pattern of the entire neuraxis. Subsequently, Wnt signaling becomes important in individual brain regions. In the caudal forebrain, the thalamus is an area that shows enriched expression of ligands, receptors and mediators of the canonical Wnt signaling pathway. Wnt3 and Wnt3a mark the *MDO *and the dorsal part of the thalamus in fish, an expression pattern that is conserved in the vertebrate lineage as recent work has demonstrated that both ligands are similarly expressed in the embryonic thalamus of the chick [14] and mouse [34]. During thalamic complex development, however, a comprehensive picture of the function of Wnt signaling is still lacking and only recently individual aspects have begun to be elucidated. Inhibition of canonical Wnt signaling by Dkk-1 transforms the thalamus into pre-thalamus during the early regionalization phase [35]. Furthermore, it has been shown that the pre-thalamus marker Lhx5 can activate the expression of the extracellular Wnt inhibitor sFRP1a and sFRP5 [22]. These data suggest that canonical Wnt signaling is required for thalamus development, whereas the development of the pre-thalamus requires inhibition of canonical Wnt signaling.

The canonical Wnt signaling pathway plays a pivotal role in mediating the clustering of cells. The key effector of the Wnt pathway, β-catenin, promotes adhesiveness by binding to the transmembrane adhesion molecule cadherin [36,37]. Recently, a member of this group, the Protocadherin 10b (Pcdh10b, formerly known as OL-protocadherin) has been shown to modulate cell adhesion in the thalamic complex [15]. Stabilization of ß-catenin leads to a broadening of the expression domain of *pcdh10b *whereas inhibition of Wnt signaling blocks *pcdh10b *expression. Hence, alteration of *pcdh10b *expression in the thalamus leads to an intermingling of thalamic cells with the neighboring brain areas, predominantly with the pretectum. Furthermore, Wnt signaling seems to play a crucial role in thalamic neurogenesis as post-mitotic neurons express Wnt specific target genes such as *lef1 *[38] and these markers have been shown to be activated by Wnt signaling during late thalamic maturation [15].

The foregoing descriptions notwithstanding, our knowledge of the requirement for Wnt signaling for the formation of the *MDO *is still fragmented. Reduced Wnt signaling activity in the Lrp6 -/- knockout mouse led to a reduction of the *MDO *and thalamus [39], and the expression of thalamic transcription factors, such as Gbx2, is severely down-regulated in these mice, suggesting that Lrp6-mediated Wnt signaling is required for proper thalamus development. However, organizer markers, such as Wnt3 and Shh, are similarly down-regulated. These data support our observation that lack of Wnt signaling leads to a malformation of the organizer tissue in zebrafish. Interestingly, we identified a narrow window of four hours during somitogenesis, which is sufficient to maintain the organizer anlage in zebrafish. This time point correlates with the expression dynamic of both ligands as co-expression of Wnt3 and Wnt3a in the organizer can only be observed between the 10 and the 16 somite stages (Figure 2). In light of our data the defects observed in the Lrp6-/- mouse thalamus may be interpreted as a dual phenotype, (i) disruption of the *MDO *and (ii) misspecification of thalamic cells - both due to a lack of Wnt signaling.

### Fez and Irx are able to suppress *MDO *competence in pre-thalamus and thalamus

Interestingly, we find that the expression of pre-thalamic markers, such as *fezf2 *(Figure 5) and *lhx5 *(Figure 6), as well as thalamic markers, such as *irx1b *and *otx2 *(Figure 6), are not affected by the abrogation of Wnt signaling during somitogenesis. Although the *MDO *area disappears, the size of the surrounding territories is maintained. This suggests that development of the primordia of pre-thalamus, *MDO *and thalamus are largely independent at this early stage. Indeed, cell lineage restriction operates at the borders of the organizer [13,40]. However, by simultaneous knockdown of Fezf2 function in the pre-thalamus or Irx1b function in the thalamus in Wnt deficient embryos, we were able to rescue the formation of the organizer. We found that the territory of the caudal forebrain and midbrain is similarly small in Wnt3/Wnt3a double morphant embryos compared to the triple morphant embryos. Therefore, we propose a comparable increased rate of apoptosis. However, we found that both pre-thalamus and thalamus are competent to form the *MDO *organizer. However, they lose their competence for organizer induction by expression of the transcription factors, Fez or Irx. Indeed, both transcription factors have been characterized by their pivotal repressive function during neural development [41-43]. For example, Irx2 restricts the *MHB *organizer primordium by suppression of the competence in the cerebellum to adopt *MHB *organizer fate [42]. Consequently, a dominant-negative version of Irx2 leads to ectopic induction of the organizer. Although the *MHB *organizer is characterized by the expression of several Wnt ligands, the relation between Wnt signaling and Irx function is unclear during organizer formation.

Thus, we may conclude that canonical Wnt signaling is required for maintenance of the organizer primordium and, subsequently, for the formation of the entire thalamic complex.

### Wnt signaling and apoptosis

Wnt signaling has been suggested as a crucial survival factor in many contexts. In Drosophila, patches of cells that are deficient in Wg signal transduction are progressively eliminated by apoptosis [44,45]. In vertebrates, Wnt signaling has been suggested as an important external trigger for proliferation of stem cell and cancer cells [27]. During the development of the central nervous system, stabilization of ß-catenin in neural precursors leads to enlarged brains with increased cerebral cortical surface area and folds, suggesting that Wnt signaling can regulate cerebral cortical size by controlling the generation of neural precursor cells [46]. Consistently, reduction of ß-catenin signaling leads to reduction of central nervous tissue as the neuronal precursor population is not maintained [47]. Here, we show that blockade of canonical Wnt signaling leads to specific cell death in the *MDO*. Recently, it has been suggested that Morpholino oligomeres *per se *may induce Tp53mediated apoptosis [48,49]. However, we provide evidence that apoptosis observed in compound morphant embryos is due to a specific loss of Wnt3/Wnt3a function. First, we observed locally enriched apoptosis within the organizer tissue, but the surrounding areas are unaffected. Second, we observed a similar apoptotic phenotype after treatment with the small molecule inhibitor IWR1 and the organizer is similarly reduced in embryos with ectopically induced Dkk1 expression. Third, we were able to restore the organizer in double morphant embryos by treatment with the Wnt agonist BIO and we rescue the organizer in embryos treated with IWR1 by simultaneously blocking Tp53-mediated apoptosis. Taking these arguments together, we conclude that Wnt3 and Wnt3a are required for protecting the organizer tissue from Tp53-mediated apoptosis. Consistently, our findings are supported by a recent observation in cancer cells suggesting that Tp53-mediated apoptosis acts in a negative feedback loop with Wnt signaling [50].

## Conclusion

In summary, we show that canonical Wnt signaling is required for regionalization of the caudal forebrain. Alteration of the canonical Wnt signaling pathway leads to apoptosis of the *MDO *primordium and subsequently to a mis-specification of the entire thalamic complex (Figure 9). We suggest that Wnt3 and Wnt3a are the crucial Wnt ligands, which are required between 10 h and 14 h to maintain the *MDO *anlage by protecting the cells from Tp53mediated apoptosis. Thus, by determining *MDO *fate and thalamic compartition, Wnt3 and Wnt3a control the development of the organizer of the major relay station in the brain - the thalamus.

## Methods

### Maintenance of fish

Breeding zebrafish (*Danio rerio*) were maintained at 28°C on a 14 h light/10 h dark cycle [51]. To prevent pigment formation, embryos were raised in 0.2 mM 1-phenyl-2-thiourea (PTU, Sigma, St. Louis, MO 63103 USA) after 24 hpf. The data we present in this study were acquired from analysis of KIT wild-type zebrafish AB2O2 as well as the transgenic zebrafish line *Shh::RFP *[25], *Nkx2.2::GFP *and *masterblind *mutant line carrying a mutation in *axin1 *[21].

#### Functional analysis

Transient knock-down of gene expression was performed as described in [52]. We used the following Morpholino-antisense oligomeres (MO, Gene Tools, Philomath, OR 97370 USA) at a concentration of 0.5 mM: *wnt3 *MO (5'-GATCTCTTACCATTCGTCCTGC-3'), 0.25 mM *wnt3a *MO [53], *irx1b *MO [54], *fezf2 *MO [31], and *Tp53 *MO [55]. The injection of MO oligomers was performed into the yolk cell close to blastomeres at one-cell or two-cell stage.

To manipulate Wnt signaling *in-vivo*, we used BIO [20]; (2'Z,3'E)-6-Bromo-indirubin-3'oxime, TOCRIS Bioscience, Minneapolis, MN 55413 USA) or IWR-1 [18]; (Sigma) as pharmacological agonist and antagonist of the Wnt signaling pathway. For Wnt signaling analyzes, embryos were dechorionated and incubated with 4 μM of BIO in 1% dimethyl sulfoxide (DMSO), 40 μM IWR-1 in 0.2% DMSO or with 1% DMSO only at given time points. Heparin-coated acrylic beads (Adar Biotech, Rehovot 76360 Israel) were prepared as described previously [56]. The beads were coated with recombinant Wnt3a protein (R&D Systems, Minneapolis, MN 55413 USA) and implanted dorsally into the region of the presumptive *MDO *of wild-type embryos at the 10 hpf. HS-Dkk1-GFP DNA [57] was injected into one-cell stage embryos. A 15-minute heat shock treatment at 42°C was performed at 10 hpf. All treated embryos were incubated at 28°C and fixed at 28 hpf.

#### Staining procedures

Prior to staining, embryos were fixed in 4% paraformaldehyde/PBS at 4°C overnight for further analysis. Whole-mount mRNA *in situ *hybridizations were performed as described in [58]. The expression pattern and/or antisene RNA probes have been described for *wnt3 *(formerly known as *wnt3l*) and *wnt3a *[59], *shh (shh-a) *[60]), *shh-b *[61], *ptc1 *[62], *axin2 *[63], *lhx5 *[64], *irx1b *[54], *otx1l *and *otx2 *[65], *neurog1 *[66], *fezf2 *[67].

SDS-PAGE/Western blot analysis was performed with polyclonal antibodies to detect Wnt3 (GTX105679, Acris Antibodies, San Diego, CA 92121 USA) and Wnt3a (ab28472, Abcam, Cambridge, CB4 0FL UK) and a monoclonal antibody against PCNA (sc-56, Santa Cruz Biotechnology, Santa Cruz, CA, USA) as loading control, respectively.

#### Image acquisition

Prior to imaging, embryos were de-yolked, dissected and mounted in 70% (v/v) glycerol/PBS on slides with cover slips. Images were taken on an Olympus SZX16 microscope equipped with a DP71 digital camera by using the imaging software Cell A. For confocal analysis, embryos were embedded for live imaging in 1.5% low-melting-point agarose (Sigma) dissolved in 1× Ringer's solution containing 0.016% tricaine at 48 hpf. Confocal images stacks were obtained using the Leica TCS SP5 confocal laser-scanning microscope. We collected a series of optical planes (z-stacks) to reconstruct the imaged area. Rendering the volume in three dimensions provided a view of the image stack at different angles. The step size for the z-stack was usually 1 to 2 μm and was chosen upon calculation of the theoretical z-resolution of the 40× objective. Images were further processed using Imaris 6 (Bitplane AG, CH-8048, Zurich Switzerland).

## Abbreviations

Cer: Cerebellum; cFb: Caudal forebrain; DMSO: Dimethyl sulfoxide; Eth: Epithlamus; HyTh: Hypothalamus; Mb: Midbrain; MDO: Mid-diencephalic organizer; MHB: Midbrain-hindbrain boundary; PBS: Phosphate-buffered saline; PTh: Pre-thalamus; PTh-p: Pre-thalamus primordium; Tel: Telencephalon; Th: Thalamus; Th-p: Thalamus primordium; ZLI: Zona limitans intrathalamica.

## Competing interests

The authors declare that they have no competing interests.

## Authors' contributions

BM, SW and JP carried out the molecular genetic studies displayed in this manuscript. QC carried out the immunoassays. SS, CH and GD conceived the study, and participated in its design and coordination. SS guided the project and wrote the manuscript. All authors read and approved the final manuscript.
